# Exploring the Analytical Complexities in Insect Powder Analysis Using Miniaturized NIR Spectroscopy

**DOI:** 10.3390/foods11213524

**Published:** 2022-11-05

**Authors:** Jordi Riu, Alba Vega, Ricard Boqué, Barbara Giussani

**Affiliations:** 1 Universitat Rovira i Virgili, Department of Analytical Chemistry and Organic Chemistry, Carrer Marcel·lí Domingo 1, 43007 Tarragona, Spain; 2 Dipartimento di Scienza e Alta Tecnologia, Università degli Studi dell’Insubria, Via Valleggio, 9, 22100 Como, Italy

**Keywords:** edible insect powder, miniaturized instrumentation, measurement strategies, near-infrared spectroscopy, classification, prediction of macronutrients

## Abstract

Insects have been a food source for humans for millennia, and they are actively consumed in various parts of the world. This paper aims to ascertain the feasibility of portable near-infrared (NIR) spectroscopy as a reliable and fast candidate for the classification of insect powder samples and the prediction of their major components. Commercially-available insect powder samples were analyzed using two miniaturized NIR instruments. The samples were analyzed as they are and after grinding, to study the effect of the granulometry on the spectroscopic analyses. A homemade sample holder was designed and optimized for making reliable spectroscopic measurements. Classification was then performed using three classification strategies, and partial least squares (PLS) regression was used to predict the macronutrients. The results obtained confirmed that both spectroscopic sensors were able to classify insect powder samples and predict macronutrients with an adequate detection limit.

## 1. Introduction

Over the last decade, rapid population growth and the problems of undernourishment, mainly in underdeveloped countries, are creating the need to find new and sustainable food sources, and a new perspective is being demanded to address this issue. Insect-based products seem to match this priority [1,2], by incorporating important nutrients in the human diet such as protein, vitamins, and minerals [3,4].

Unfortunately, even if the consumption of edible insects started nearly 7000 years ago and many insects have been eaten worldwide [5], consumers in many countries exhibit food neophobia toward products with insect-based ingredients [6], and legislation on the distribution of insect and insect-based products for human consumption does not go hand in hand in all regions of the world [7]. In Europe, for example, edible insects are novel foods according to the Regulation (EU) 2015/2283 and they must be included in the list of authorized novel foods before being placed in the market. The first species to be included in this list was *Tenebrio molitor*, declared safe by the European Food Safety Authority (EFSA) in January 2021. However, if edible insect powders have previously reached national markets based on national regulations, they can still be found in these national markets. The overlapping of the new European regulation (and the list of novel foods) and previous national regulations, mean that it is difficult nowadays to find a wide variety of edible insect powders in Europe.

The diffusion of a new type of food requires new analytical strategies for its analysis, and to control the quality of such food. This paper tries to address this problem, proposing a rapid and low-cost methodology for the modern insect-based food industry and consumers. A few reports can be found in the literature that analyze insect-based products [7,8,9], showing that NIR and ATR-FTIR (attenuated total reflectance—Fourier transform infrared) spectroscopies may be good options for the analysis of these foods. The possibility to discriminate insect powder samples using low-cost and portable techniques, such as miniaturized spectrometric devices working in the near-infrared (NIR) region of the electromagnetic spectrum, is here explored, and, to our knowledge, presented for the first time.

The choice of miniaturized NIR spectroscopy arises from several considerations, since this technology has proved its suitability in many applications, and has solved analytical problems relevant to food analysis [10,11,12,13].

NIR miniaturized spectroscopy is non-destructive, fast, cheap, and almost no chemicals are needed, all of which greatly reduce the impact the technique and the analysis have on the environment. It is portable and it can be used directly on the field or in the plant, and by the end-user without any sample pretreatment, once the necessary chemometric models for data analysis have been developed. The use of multivariate analysis goes hand in hand with NIR spectroscopy.

Most currently available NIR miniaturized sensors are not designed to analyze powder samples [10], and the first step of this investigation was therefore the optimization of the analytical strategy, including the design and fabrication (using a 3D printer) of a specific homemade cell for the measurements. Once the method of analysis was optimized from the technological point of view, there was another factor that has been considered: particle size. Since the typical measurement modes for most current miniaturized NIR instruments are reflectance or transflectance, particle size is an important factor that affects light scattering, and, thus, affects the obtained analytical signal [14]. There are various reports discussing the relationship between particle size and the spectroscopic data in the NIR region [15,16].

This research explores the analytical complexities encountered when analyzing insect powder samples and proposes a reproducible way for their analysis without any pretreatment using two different portable NIR spectrometers (SCiO, Consumer Physics, and NeoSpectra Micro Development Kit, Si-Ware), with quite different characteristics and modes of operation.

Five commercially available insect powder samples were purchased, and their differentiation and classification were carried out using principal components analysis (PCA), partial least squares 2-discriminant analysis (PLS2-DA), and linear discriminant analysis (LDA) algorithms. Finally, PLS regression was used to predict the content of macronutrients in the samples.

## 2. Materials and Methods

### 2.1. Materials and Instruments

A SCiO spectrometer (version 1.2—Consumer Physics, Herzliya, Israel) and a Neospectra Micro Development Kit (MDK) device (Si-Ware, Cairo, Egypt) were used for the insect powder analysis.

The SCiO is a miniaturized NIR device that uses a molecular sensor with dimensions of 67.7 mm × 40.2 mm × 18.8 mm and weighs 35 g. It works in the wavelength range of 740 to 1070 nm, and it is controlled by a smartphone (Android and IOS) through the application ‘SCiO Lab’ via Bluetooth. The default scan time for SCiO measurements is between 2 and 5 s, and it cannot be set manually. The data recorded are stored in the cloud. The resolution of the SCiO device is not available from the manufacturer [13].

The NeoSpectra MDK consists of a monolithic microelectromechanical system (MEMS) Michelson interferometer and a single InGaAs photodetector. The dimensions of the device are 32 mm × 32 mm × 22 mm and it weighs 17 g. The wavelength range is 1350 to 2558 nm with a resolution of 16 nm. The NeoSpectra Micro is connected to a Raspberry Pi single-board computer that acts as a host and allows connection via a universal serial bus (USB) to a laptop. The software (Windows and Linux) allows the user to set a limited number of parameters, such as the scan time, run mode (single or continuous), or data interpolation in each spectrum collected. The NeoSpectra has to be calibrated each time the software is started. The scan time was optimized to 5 s.

A background (using a 99% reflectance material) was performed before any analytical session as suggested by the manufacturers for both sensors. In the case of the SCiO, the reflectance material is included on the back side of the cover. The analytical sessions started after 20 min warming for the NeoSpectra MDK sensor.

Reflectance mode analysis was employed for both instruments. A homemade cell was used as the sample holder (a description is given in the section ‘Optimization of the instrumental Setup’ of this paper). A MakerBot Replicator^®^ 2 Desktop 3D Printer (MakerBot Industries, New York, NY, USA) was used to fabricate the holder lateral sides, while in the intermediate bottom of the cell, interchangeable coverslips of 22 mm × 22 mm pieces of borosilicate glass of hydrolytic class 1 with a thickness of 0.13–0.17 mm (Knittel Glass, Bielefield, Germany) were used.

A coffee grinder (Black+Decker BXCG150e, Oliana, Spain) was employed to grind the insect powder samples.

### 2.2. Samples

Five types of commercial edible insect powders were purchased. The insect type, the producer or importer, and the label/class assigned to these types are:

- *Acheta domesticus* (Nimavert, Harelbeke, Belgium), class A1
- *Acheta domesticus* (Crunchy Critters, Derby, UK), class A2
- *Tenebrio molitor* (Nimavert, Harelbeke, Belgium), class T
- *Alphitobius diaperinis*, (Entofood, Emerlo, The Netherlands), class AD
- *Locusta migratoria*, (Entofood, Emerlo, The Netherlands), class L

Details of the samples reproduced from the commercial labels are reported in Table S1 in the Supplementary Materials.

The samples were analyzed without any sample pretreatment and after grinding (except for insect powder A2, which was a very fine powder and was therefore not further ground). Sample bags were only opened immediately before the analysis, and after opening, the sample bags were stored under a nitrogen atmosphere and in a temperature-controlled (4 °C) and dark environment to prevent any unwanted and unpredictable changes. Stored samples were analyzed when they reached ambient temperature. Measurements were carried out at ambient temperature and without any special precautions. An instrumental background was performed prior to any analytical session. Fifteen aliquots of each bag were sampled and analyzed in three independent analytical sessions (on different days), except for A2. Three bags of sample A2 were purchased and five aliquots for each bag were sampled—a bag for each analytical session. In any session, all the insect powders were investigated. In all sessions, data were collected with both spectrometers.

### 2.3. Statistical Data Analysis

Calculations were performed using PLS Toolbox 9.0 (Eigenvector Inc., Manson, WA, USA) running under the Matlab 2021a (Mathworks Inc., Natick, MA, USA) environment.

Two data matrices were built, one for signals recorded with the SCiO sensor (range 740 to 1040 nm, 331 total wavelengths) and one for signals obtained with the NeoSpectra MDK (range 1350 to 2558 nm, 134 total wavelengths). The average of five spectra recorded for each insect powder sample, collected after resampling, were used to build the **X** matrices.

PCA was used for exploratory data analysis. Classification of the different types of insect powder was carried out following three strategies: (1) one-class classification using T^2^ and Q statistics calculated in the PCA models built for each class, (2) classification using PLS2-DA, and (3) classification using LDA. PLS regression was employed to predict major components in the insect powder samples.

Several spectral preprocessing techniques were evaluated to improve classification and prediction model performances. Standard normal variate (SNV), multiplicative scatter correction (MSC), Savitzky-Golay smoothing (from 7 to 25 smoothing points), and derivatives (first and second order) were tested. Prior to any model calculation, data were mean-centered. In the case of PLS and PLS2-DA, the Venetian blinds method (with 10 data splits and 4 samples per blind) was used for cross-validation of the training set.

Samples were analyzed on three different days (three independent analytical sessions). Calibration samples were analyzed during the first two analytical sessions. Validation samples were analyzed in the third analytical session. For A2, three different bags were available: samples from two bags were used to calibrate the model, and samples from the third bag were employed to validate the model. Ground samples were analyzed in independent sessions from non-ground samples. The reason for performing measurements on different days was to capture the between-day variability, if present, and to obtain a validation set of samples (external validation [17]) as independent as possible from the calibration set.

## 3. Results and Discussion

### 3.1. Optimization of the Instrumental Setup

In this section, the instrumental setup for the NeoSpectra MDK and SCiO will be respectively discussed.

#### 3.1.1. NeoSpectra MDK

The NeoSpectra MDK sensor is designed to be used in contact with the sample and there is no commercially available sample holder for powder samples. For this reason, a homemade cell or sample holder was optimized to:

- allow for reproducible reflectance analysis,
- cover the entire measurement window,
- and allow for easy cleaning of the cell.

Concerning the last point, some of the insect powders appeared to be oily. This suggested using an easily washable or economically disposable material such as the coverslips to make the lower part of the cell (the part that is in contact with the measurement windows). The coverslip does not affect the spectra as already observed [18], is a low-cost material, and can be easily removed from the cell and cleaned or eventually replaced before any analysis. Since coverslips were replaced in every measurement, no chemicals were used.

**Figure 1:**
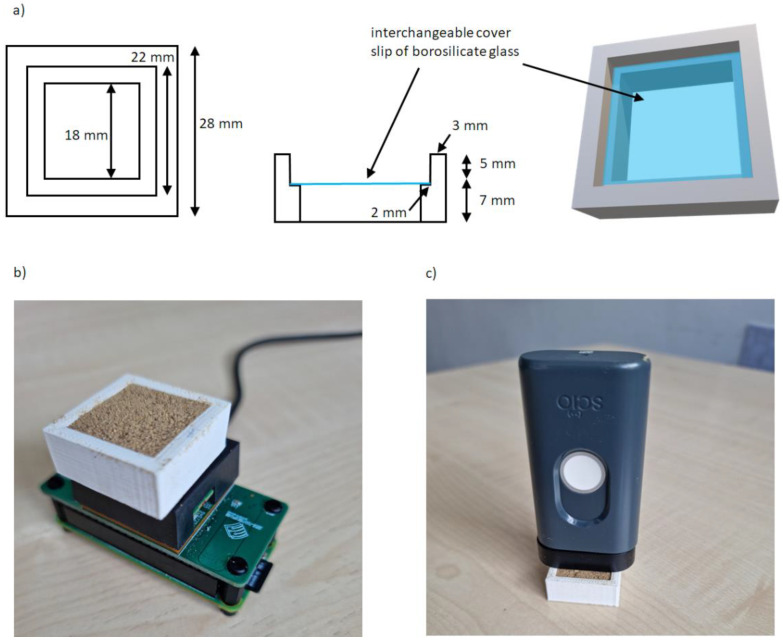
(**a**) homemade cell for the NeoSpectra MDK and SCiO NIR devices, (**b**) experimental configuration using the homemade cell for the NeoSpectra MDK measurements, (**c**) experimental configuration using the homemade cell for the SCiO measurements.

Accurate length measurements were taken to build a cell able to cover the whole measurement window and to fit perfectly into the sensor to avoid any movement/shift during the analysis. The key feature to optimize was the thickness of the cell (i.e., the thickness of the sample to be analyzed). Several experiments were carried out putting in a first cell prototype different quantities of a sample (different thicknesses) and using a reflective material on the top of the sample to detect changes in the signal according to the different optical paths of the radiation. From these experiments, a cell height of 5 mm was found to allow the obtaining of reproducible spectra without any external light influence (the optimized cell was used in different light conditions without any change in spectral shape or intensity) using a sample quantity easy to manage but without excessive waste. The transflection mode of analysis was also considered but results were not satisfactory in terms of reproducibility of the spectroscopic signal: the measurement window is quite large and adding the minimum amount of sample to allow the radiation to pass through it twice and at the same time covering the entire window was not trivial. For these reasons, the external reflection mode was preferred. The same cell was tested on wheat flour and corn flour and the 5 mm height was also suitable for these other types of powder samples. A scheme of the homemade cell is depicted in Figure 1a:

After the optimization of the homemade sample holder, optimization of the scanning time was carried out.

Five seconds of scanning time was the shortest time that ensured reproducible spectra, while less time resulted in noisier spectra. Ten instrumental replicates were collected for each sample, and each sample was repositioned five times to get the final average spectrum to be considered for calculations. Figure 1b shows the experimental configuration for the NeoSpectra MDK measurements.

As previously said, samples were stored in a controlled atmosphere (nitrogen), light conditions (dark), and temperature (4 °C), to avoid unwanted changes that would have added more variability to the system. These storage conditions became necessary after observing that the samples stored in the air for several days lost moisture during the analysis with the NeoSpectra MDK.

Figure S1 in the Supplementary Materials shows the spectra of a random sample from the A1 class after being left in air for 10 days. Looking at the spectra recorded without sample repositioning (instrumental replicates), a change is clearly visible in the band around 1950 nm, corresponding to water (and also around 1450 nm, although in this region the noise is more significant) [19].

This spectral variation could be explained considering the high temperature of the measuring window, which evidently affects the sample moisture even if the sample is not in direct contact with the window (the cover slip placed at the bottom of the homemade cell lies between the measuring window and the sample). This behavior was not observed when samples were analyzed immediately after opening the sample bag, or after reaching ambient temperature after the proper storage previously described. The same behavior was observed with all types of insect powders when analyzing them with the NeoSpectra MDK.

#### 3.1.2. SCiO

The optimized homemade cell was also used in the data collection using the SCiO sensor. It should be noted that for measurements with this kind of sensor it is very important to maintain a perfectly vertical position of the sensor (in our experience, tilting affects the measurements [18]). A fixed distance combined with the use of the homemade cell facilitated this. After filling the sample holder, the sensor was placed above it. The best signal-to-noise ratio was obtained when the sensor was not in direct contact with the sample, and this was achieved by using the spacer accessory (10 mm separation) included with the SCiO. Figure 1c shows the experimental configuration for the SCiO measurements.

In this case, it was not possible to optimize the sampling time since this is automatically set by the SCiO. Five replicates with sample repositioning were registered for each insect powder aliquot and the average spectrum was used for the calculations.

### 3.2. Spectroscopic Signals

**Figure 2:**
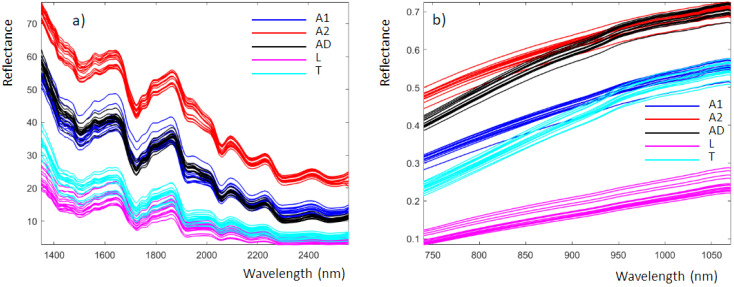
Insect powder recorded using (**a**) the NeoSpectra MDK and (**b**) the SCiO, colored according to the different classes previously defined.

Figure 2 shows the average reflectance spectra obtained from the analysis of insect powders with the NeoSpectra MDK (Figure 2a) and SCiO (Figure 2b). These data correspond to those produced by the instruments and are imported into MATLAB without any preprocessing. The NeoSpectra MDK and SCiO spectra consisted of 134 and 331 points, respectively. The spectra are colored according to the classes previously defined.

For the NeoSpectra MDK (Figure 2a), the spectra are similar to those of insect powders recently published using a benchtop NIR instrument [7]. The spectra obtained with the benchtop instrument are more detailed because of the higher performance parameters of the benchtop instrumentation compared to the miniaturized device. From Figure 2a, we can highlight the following spectral bands [7]:

- 1495 nm, N-H stretch 1st overtone, NH: amide, proteins
- 1723/1726 nm, C-H stretch 1st overtone, CH_2_: lipids
- 1924 nm, O-H stretch + O-H deformation: water
- 2054, N-H asymmetric stretch + amide II: proteins
- 2164, N-H symmetric stretch + amide II: proteins
- 2298, 2x amide I + amide III: proteins
- 2301, C-H bending: lipids
- 2468, C-H combination: lipids

The SCiO spectral bands (Figure 2b) are more difficult to analyze since they are strongly influenced by scattering, which is not surprising when using the reflectance mode for fine powders. It is worth noting that samples A1 and A2 are powders obtained by the same insect type but from different manufacturers and therefore with different chemical compositions and particle size.

### 3.3. Multivariate Statistical Analysis

#### 3.3.1. Data Preprocessing and Exploratory Analysis of Insect Powders

The average spectra obtained for each insect powder aliquot were used to build the **X** matrices (one for each instrument). Data were then preprocessed according to the characteristics of the different spectroscopic signals to obtain the highest values of explained variance in the first PCs, which corresponded to the best separation between classes in PCA models.

**Figure 3:**
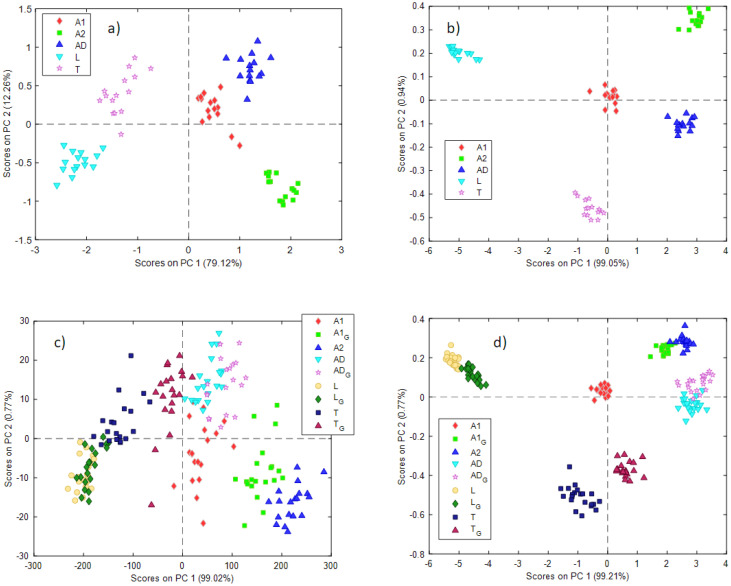
Scores plots for the two first PCs obtained with the PCA models using the (**a**) NeoSpectra MDK with non-ground data, (**b**) SCiO with non-ground data, (**c**) NeoSpectra MDK with ground and non-ground data, (**d**) SCiO with ground and non-ground data. A ‘G’ in the label indicates ground insect powder.

Data obtained from the NeoSpectra MDK gave the best results after smoothing (21 points) and the first derivative, while SCiO data did not need any preprocessing. Figure 3 shows the score plots for the first 2 PCs (Figure 3a—NeoSpectra MDK, Figure 3b—SCiO). Samples are labeled and colored according to the type of insect powder samples.

In both cases, a good separation between the different insect powder samples can be observed. The information is distributed on the two first PCs in the case of the NeoSpectra MDK data (79% PC1, 12% PC2) while PC1 dominates the variability of the samples in the case of the SCiO sensor (99%). The loading plots for the two PCA models can be found in the Supplementary Materials (Figure S2).

An interesting observation emerges from both models. Looking at the different groups of samples, they seem to have the same intrinsic variability: in other words, they occupy a comparable area in the PC1 vs PC2 score plot. As previously mentioned, aliquots of insect powder A2 (labeled as green squares in the plots) were collected from 3 different bags (5 aliquots each). A2 samples do not show any behavior according to the different bags, nor do A2 samples show a very different variability from others. No trends with respect to the three bags are observed. This suggests that the variability between different bags is comparable to the estimated variability within the same bag.

#### 3.3.2. Exploratory Analysis of Ground Insect Powders

As previously mentioned, the purchased insect powders showed different textures, some of them appearing as fine powders, and others as coarser powders. As the miniaturized NIR instruments employed in this research work in reflectance mode (as in many of the applications of miniaturized NIR sensors), the registered spectra may be affected by the physical characteristics of the samples. While this aspect is often overlooked in many studies, the influence of the sample texture on the modeling was investigated.

A dimensional homogenization of the insect powder samples was performed. All the samples except sample A2, which was a very fine powder, were treated using a commercial coffee grinder. To avoid any unwanted change in the samples (e.g., due to friction in the grinder) the insect powders were ground in four 30 s consecutive sessions, with a pause of 10 s between each session.

When building the PCA models with the ground and non-ground insect powders, data obtained from the NeoSpectra MDK gave the best results after smoothing (21 points), while SCiO data did not need any preprocessing. Very interesting results can be observed in the PCA scores obtained (Figure 3c—NeoSpectra MDK, Figure 3d—SCiO). In these models, insect powder samples after and before the grinding process are depicted (a ‘G’ in the label indicates the ground sample).

Looking at the PCA model built with NeoSpectra MDK data (Figure 3c), the ground insect powder samples lie near the raw respective samples, but the groups of samples are now rather overlapping. The most interesting results concerned insect powders A1 and A2, which are of the same insect, *Acheta domesticus*. When insect powder A1 is ground, its position in the score plot moves towards insect powder A2, which appears as a very fine powder as previously mentioned (and for this reason, it was not ground).

This has two important implications. The first is that, as expected, the spectroscopic signal depends on the physical, as well as the chemical, characteristics of the powders. The second is that using portable NIR spectroscopy and chemometric analysis, the classification of insect powders may be possible only by taking into account the granulometric characteristics of the samples.

These results are confirmed also by data collected with the SCiO sensor. In the scores plot (Figure 3d) samples before and after grinding are depicted. In this case, a good separation between the different insect powder samples is retained, even if samples T and T_G are not overlapped in the space of the two first PCs. Also, in this case, the ground A1 sample overlaps the A2 samples, confirming the encouraging results already seen for the data collected with the NeoSpectra MDK sensor.

#### 3.3.3. Classification of the Insect Powder Samples

Three different classification strategies were tested and validated. The first one is based on an unsupervised method, Hotelling’s T^2^ and Q statistics calculated in PCA models, while the other two strategies were developed using two supervised methods of classification: PLS2-DA and LDA.

For the classification of the different insect powders, we decided to focus on the type of insect, introducing in the same class the different producers and grinding levels. The aim was to develop classification models able to classify different types of insect powders regardless of their provenance or grinding level (PCA models also showed the possibility to differentiate all the samples between them).

Therefore, samples from classes A1, A2, and A1_G (*Acheta domesticus* from two different producers and with different grinding levels) were all labeled as class A. Samples from classes AD and AD_G were labeled as class AD. Samples from classes T and T_G were labeled as class T. Samples from classes L and L_G were labeled as class L.

#### One-Class Classification Based on PCA

The first type of classification was carried out using Hotelling’s T^2^ and Q statistics calculated in PCA models: independent PCA models for each class were built with the calibration samples, and the samples belonging to the other classes were projected onto these models, together with the validation samples of the considered class. Samples outside the confidence intervals defined by the T^2^ and Q statistics can be classified as samples not belonging to that class. The criterion to select the number of PCs for each PCA model was to select the minimum number of PCs that explained at least 90% of the variance. What is expected through the validation of this kind of model is that, for a certain class, validation samples belonging to the same class will lie in the inner part of the confidence intervals defined by the T^2^ and Q statistics, while samples belonging to the other classes will lie outside the confidence interval. This strategy is one of the most used in the ‘one-class classification approaches [20].

The preprocessing used for the different classes and the number of required PCs using NeoSpectra MDK data were:

- class A: smoothing (21 points), 4 PCs
- class AD: no preprocessing, 1 PC
- class L: no preprocessing, 1 PC
- class T: first derivative, 3 PCs.

**Figure 4:**
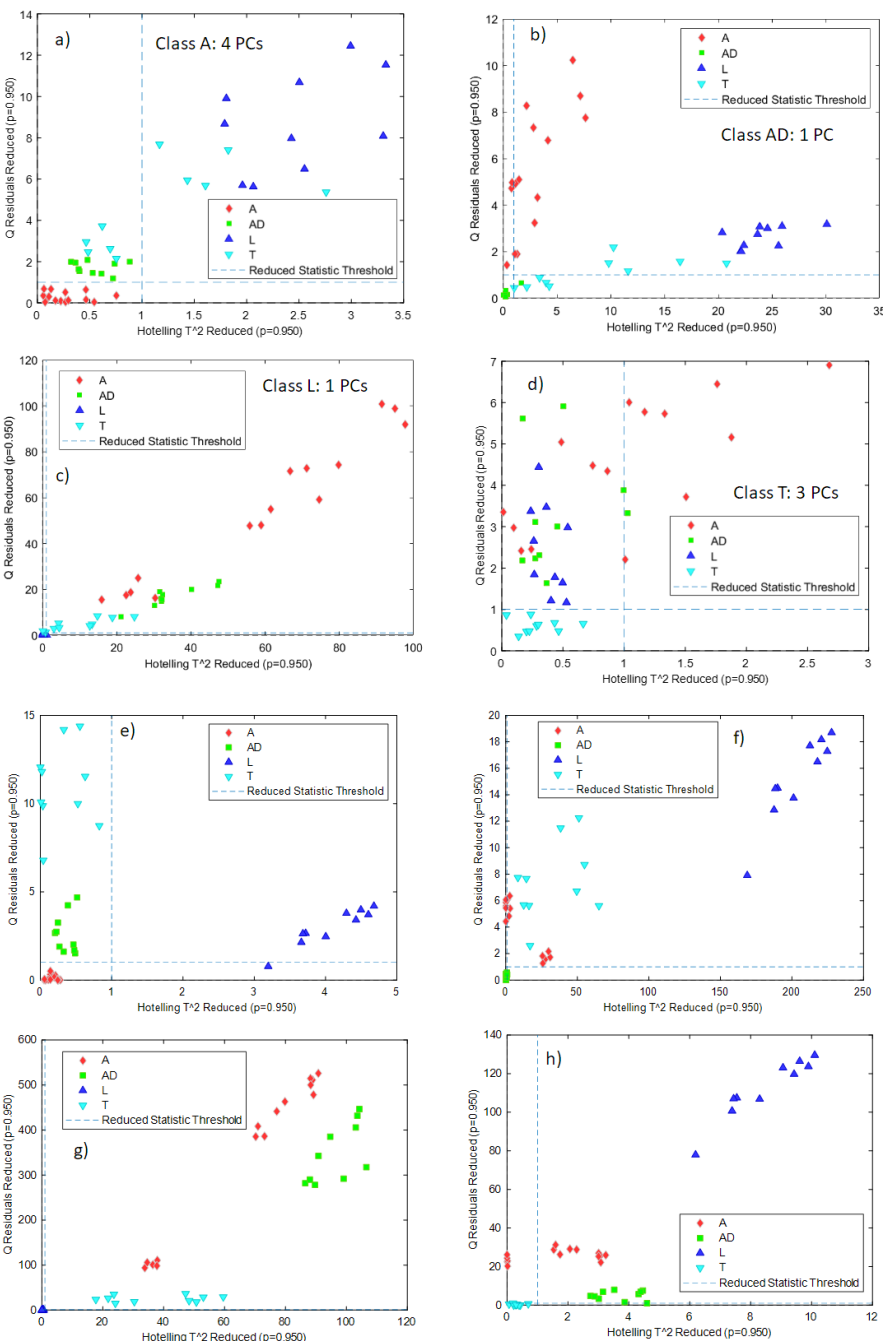
Projection of samples of other classes onto the Hotelling’s T^2^ and Q confidence intervals for the individual PCA models built for each class: (**a**) class A NeoSpectra MDK data, (**b**) class AD NeoSpectra MDK data, (**c**) class L NeoSpectra MDK data, (**d**) class T NeoSpectra MDK data), (**e**) class A SCiO data, (**f**) class AD SCiO data, (**g**) class L SCiO data, (**h**) class T SCiO data. Results are without differentiating grinding level or producer.

Figure 4a–d shows the T^2^ and Q confidence intervals (lower-left part of each subfigure defined by the dashed lines) for each one of the classes built with the NeoSpectra MDK data. For the PCA models, more than 90% of the variance was explained for classes AD and L, and more than 95% of the variance was explained for classes A and T. For all models, samples belonging to a particular class were correctly classified in that class with the exception of one sample in class AD and one sample in class L. Figure 4a–d also shows that samples belonging to other classes lie away from the region defined by the T^2^ and Q confidence intervals, showing that those samples are correctly classified as not belonging to that class. The sensitivity (the ability of the model to correctly classify samples of its class) [21] is 100% for classes A and T and 90% for classes AD and L, and the specificity (the ability of the model to correctly reject samples from other classes) is 100% in all cases. Class A needs four PCs to be modeled: it is worthwhile to recall that class A includes samples from two different producers (A1 and A2) and therefore the model needs more factors to cover the whole variability of the data.

Data obtained from the SCiO data gave the best results without any preprocessing. Figure 4e–h shows the T^2^ and Q confidence intervals for each one of the classes built with the SCiO data. In all cases, one PC was required to define the classes, and it is possible to see that all the samples belonging to that class are correctly classified and that samples from other classes lie away from the confidence intervals. The sensitivity and the specificity are therefore 100% for the four classes. Figure 4e–h shows, therefore, that each PCA model is able to correctly distinguish the insect powder samples from other classes.

#### Partial Least Squares 2—Discriminant Analysis (PLS2-DA)

A PLS2-DA classification strategy was also used to classify the insect powder samples. Data collected with the two sensors were modeled separately. In general, the PLS-DA algorithm is an evolution of PLS, which is an algorithm proposed to solve regression problems. In PLS, a data matrix **X** is correlated to a vector **y**, which is the sample property to be predicted. In PLS-DA algorithms, the vector **y** is substituted by a dummy variable consisting of only +1 and 0 denoting in-class and out-class respectively [22,23,24]. While PLS-DA works for binary classification, PLS2-DA handles multi-class problems. In PLS2-DA, thus, **Y** is a *K*-column dummy matrix, where *K* is the class number.

Ideally, in a multi-class problem, several PLS-DAs could be performed to model each class separately: each time only one class is of concern (in-class) while the other classes are grouped as out-class. The advantage of PLS2-DA is that the algorithm considers all classes simultaneously and produces only one classification model [25].

When building the PLS2-DA models, data obtained from the NeoSpectra MDK gave the best results after smoothing (15 points) and the second derivative, while SCiO data did not need any preprocessing.

Five latent variables (LVs) were needed in the PLS2-DA model with the NeoSpectra MDK data to account for 69.13% of the information in **Y**. Four LVs were needed in the PLS2-DA model with SCiO data to account for 88.50% of the information in **Y**. Table 1 shows that the NeoSpectra MDK sensitivities and specificities for all classes are in all cases above 0.9, except in the specificity for the test set in class T. SCiO can correctly classify all the samples in all the groups in both the training and test set, with sensitivities and specificities of 1 in all cases.

For comparison purposes, to the best of our knowledge PLS2-DA has not been applied to classification in food analysis using miniaturized NIR instruments, but PLS-DA has been used in other related studies. For instance, considering food samples, different coffee samples were successfully classified (sensitivities and specificities ranging from 0.84 to 1 for the test set) [26], adulterated and non-adulterated paprika samples were discriminated with specificity greater than 0.9 [27], different types of ground and sieved teas were classified with accuracies of 86% [28] and 65% [29], and pure and adulterated wheat flours were distinguished with specificity and sensitivity of 1 [30]. More details can be found in very recently published reviews on the field ([10,31]).

#### Linear Discriminant Analysis (LDA)

To show the usefulness of miniaturized NIR instruments for the classification of edible insect powders, we also tried another classification method (LDA) not based on the decomposition of data into latent variables. When applying LDA, data obtained from the NeoSpectra MDK gave the best results after smoothing (19 points), while SCiO data did not need any preprocessing. In all cases for all four classes (A, AD, L, and T) and the training and test sets in both NeoSpectra MDK and SCiO, the sensitivities and specificities were all 1.

LDA has not been applied as widely as PLS-DA for the classification of food samples using miniaturized NIR instruments, although some studies have been performed. For example, nitrogen-based adulterants in whey protein powder were classified with accuracies of 80% [32].

#### Prediction of Macronutrients

One of the major advantages of using chemometric data analysis is that from the same dataset several different models can be developed, depending on the specific purpose. The same spectra recorded on the insect powder samples were used to predict their major constituents.

Protein, fatty acids, carbohydrates, and fiber contents in the investigated samples (no pretreatment) were predicted using PLS regression. Spectra recorded with the NeoSpectra NDK and the SCiO sensors were used as the **X** matrix, while the values reported on the bags (declared by the producers) were used as the **y** values. For each macronutrient, a model was performed by optimizing data preprocessing and choosing the best compromise between model parsimony and prediction error. Samples were divided exactly as in the classification models: samples coming from the two first analytical sessions were used to calibrate the models, while samples analyzed in the third session were used to validate the model (except for sample A2, which was treated as for the previous models).

In all cases, the best model was chosen as the best compromise between the lower root mean square error of prediction (RMSEP) for the test set, the higher R^2^_pred_ value of the regression line between the predicted and the measured values (R^2^ corresponds to the square of the correlation coefficient of the regression line), and the smaller number of factors. Table 2 summarizes the main results obtained in the prediction of the nutritional properties. Table 2 also shows the different values of the root mean square error of cross-validation (RMSECV) obtained when building the model with the calibration set and the R^2^_CV_ value of the regression line between the predicted and the measured values of the training set in the different cross-validation steps. The different regression lines between the predicted and the measured values are shown in the Supplementary Materials (Figures S3 and S4). For those nutritional components in which the lower value of the range was closer to 0 (carbohydrates and fiber), the multivariate limits of detection (LOD) were also calculated. The multivariate limits of detection were calculated using the approximate expression for the sample-specific standard error of prediction (SEP) [33,34].

For the PLS models built with the SCiO data, no preprocessing was needed. For the NeoSpectra data, the best combinations of data preprocessing were:

- proteins: first-order polynomial smoothing with 15 points followed by the Savitzky–Golay first derivative.
- fatty acids: zero-order polynomial smoothing with 21 points followed by the Savitzky–Golay second derivative.
- carbohydrates: first-order polynomial smoothing with 11 points followed by SNV
- fiber: second-order polynomial smoothing with 21 points followed by the Savitzky–Golay first derivative.

To check the performance of these prediction models, two parameters, the ratio of performance to deviation (RPD) and range error ratio (RER), were used. Both RPD and RER are dimensionless parameters, where RPD is calculated as the ratio of the standard deviation of the reference values of the samples in the validation set to the RMSEP, and RER is calculated as the ratio of the range of the reference values in the validation set to the RMSEP [35]. Higher RPD and RER values suggest models with increasing accuracy. Calibration models with RPD ≥ 5 and/or RER ≥ 10 are evaluated as suitable for quality control, those with RER ≥ 15 are suitable for good calibration for quantification, and those with RPD ≥ 8 are suitable for applied research [36]. RPD values between 2.5 and 5 are satisfactory for screening [37]. Table 2 shows that all SCiO predictions are very accurate and suitable for applied research, with only the predictions for fatty acids less accurate but close to the quality control level. The predictions obtained with the NeoSpectra are less accurate, with the best prediction being that of fatty acids. It is worth noting that all the values are above the calculated limits of detection.

PLS for macronutrient prediction has been extensively used in food matrices analyzed by miniaturized NIR instruments. Details can be found in very recently published reviews on the field [10,31]. Most of these papers for the prediction of macronutrients calculate the RMSECV and RMSEP values, and since these values are dependent on the concentration range, the comparison is not straightforward. It is interesting, therefore, to compare the values of our prediction models in Table 2 with some published papers using the RPD and RER values, because the comparison is direct, and is not affected by the concentration range. The use of RPD and RER values is not widely used, but some published papers for the prediction of macronutrients using benchtop NIR instruments report them. For instance, the prediction of micro- and macro-nutrients was carried out in soybean crops with RPD values ranging between 1.03 and 4.33 [38], or prediction of the chemical composition in aquafeed samples used for nutrition on various aquatic species reported RPD values mostly between 1.2 and 10.9, with only an excellent RPD value of 15.6 [39]. The reported RPD values in this manuscript (included in Table 2) are globally better (especially for the PLS models using SCiO) than the RPD values reported in the above-mentioned reviewed papers, especially considering that the models reported in this manuscript have been built using miniaturized NIR instruments.

## 4. Conclusions

This manuscript describes for the first time the application of miniaturized NIR instruments to the classification of edible insect powders. The two NIR instruments used have proved their usefulness in the classification of different insect powders, regardless of their provenance or grinding degree. Depending on the type of samples used, measuring with miniaturized NIR instruments may be a challenging task to optimize the instrumental signal, and a homemade cell using a 3D printer was designed and built for this task.

Three different methods of classification were applied to successfully discriminate the different classes of insect powders. Very good results were obtained using all three methods. The results suggest that both devices have the potential to be used, e.g., in the quality control phases in the insect food industry. At the time of this study, obtaining insect powders was difficult due to changes in European regulations mentioned in the introduction. The new regulation requires that edible insects be included in the list of authorized novel foods before being introduced to the market, which only allowed access to a limited number of insect powders that had previously reached national markets based on national regulations. The goal of this study was the development of an analytical procedure for the analysis (including classification and prediction of macronutrients) of insect powders using miniaturized NIR spectroscopy, and this goal has been pursued with the currently available insect powders. Future studies dealing with a larger set of samples should be carried out to validate it.

Prediction of the macronutrients also gave very encouraging results. It should be noted that values declared by the producers were used as reference values, and the precision of this data influenced the quantification errors.

The exploitation of miniaturized NIR instruments is a field of very rapid expansion, and they are increasingly being applied to a wide variety of analyzes in many different environments and situations. In all cases, the operational conditions must be optimized to obtain the best instrumental signal and, therefore, the best results. This may involve intensive work optimizing and developing suitable measurement conditions considering the specific properties of the samples to be measured and the specific characteristics of the instruments used. This optimization process may require extensive experience in analytical measurement and the use of chemometric methods, although once the chemometric methods have been developed they can be used by non-experienced users such as final consumers.

## Figures and Tables

**Table 1:** Classification of different classes of insect powders applying PLS2-DA classification to NeoSpectra MDK and SCiO data. The values corresponding to the training set are obtained using CV.

		Training Set Sensitivity	Training Set Specificity	Test Set Sensitivity	Test Set Specificity
NeoSpectra MDK	A	1	0.983	0.967	0.983
	AD	1	0.986	1	0.986
	L	1	1	1	0.986
	T	0.950	0.900	0.900	0.871
SCiO	A	1	1	1	1
	AD	1	1	1	1
	L	1	1	1	1
	T	1	1	1	1

**Table 2:** Best prediction models obtained for the macronutrients in insect powder samples. LV = latent variables. Range, RMSECV, RMSEP, and LOD are expressed as g/100 g insect powder. The component values are as they are expressed in the commercial labels.

	Component	Range	LVs	R^2^_CV_	RMSECV	R^2^_pred_	RMSEP	RPD	RER	LOD
NeoSpectra MDK	Proteins	50–66	2	0.852	1.99	0.870	2.00	2.64	8.00	-
	Fatty acids	21.5–31.6	3	0.925	1.02	0.935	1.00	3.80	10.11	-
	Carbohydrates	1.6–6.7	5	0.810	0.80	0.745	1.03	1.81	4.95	1.54
	Fiber	3.3–8.5	3	0.708	1.05	0.709	1.10	1.80	4.73	1.98
SCiO	Proteins	50–66	2	0.977	0.78	0.987	0.59	8.95	27.12	-
	Fatty acids	21.5–31.6	7	0.862	1.39	0.940	1.04	3.65	9.71	-
	Carbohydrates	1.6–6.7	4	0.944	0.43	0.992	0.26	7.18	19.62	0.82
	Fiber	3.3–8.5	4	0.981	0.26	0.992	0.18	11.01	28.89	0.50

## Data Availability

No new data were created or analyzed in this study. Data sharing is not applicable to this article.

## Supplementary Materials

The following supporting information can be downloaded at: https://www.mdpi.com/article/10.3390/foods11213524/s1, Figure S1: 10 instrumental replicates of a random A1 sample without sample repositioning; Figure S2: Loading plots for the two first PCs obtained with the PCA models using the (a) NeoSpectra MDK and (b) SCiO data; Figure S3: Regression lines between the predicted and measured values for the different PLS models made with the NeoSpectra data. (a) protein, (b) fatty acids, (c) carbohydrates, and (d) fiber; Figure S4: Regression lines between the predicted and measured values for the different PLS models made with the SCiO data. (a) protein, (b) fatty acids, (c) carbohydrates, and (d) fiber. Table S1: Macronutrients of the samples analyzed, and labels used, in the manuscript. The content in proteins, fatty acids, carbohydrates and fiber correspond to g per 100 g of insect powder.
