# Supplementary material for: Exploring the Analytical Complexities in Insect Powder Analysis Using Miniaturized NIR Spectroscopy
Source: Foods. 2022 Nov 5;11(21):3524. doi: 10.3390/foods11213524 (PMC9659064; doi:10.3390/foods11213524)
Supplement: Supplementary file 1 [file foods-11-03524-s001.zip › foods-1957600-supplementary.pdf]

Supplementary information

Table S1 – Macronutrients of the samples analyzed and labels used in the manuscript. The content in proteins, fatty acids, carbohydrates and fibre correspond to g per 100 g of insect powder.

Insect type	Label	Producer or importer	Proteins (g)	Fatty acids (g)	Carbohydrates (g)	Fibre (g)
Acheta domesticus	A1	Nimavert, Harelbeke, Belgium	57.3	21.5	2.7	4.9
Acheta domesticus	A2	Crunchy Critters, Derby, UK	66	25.5	2	3.5
Tenebrio molitor	T	Nimavert, Harelbeke, Belgium	50	30.8	6.7	3.3
Alphitobius diaperinis	AD	Entofood, Emerlo, The Netherlands	59.6	28.7	2.7	3.7
Locusta migratoria	L	Entofood Emerlo, The Netherlands	56.3	31.6	1.6	8.5

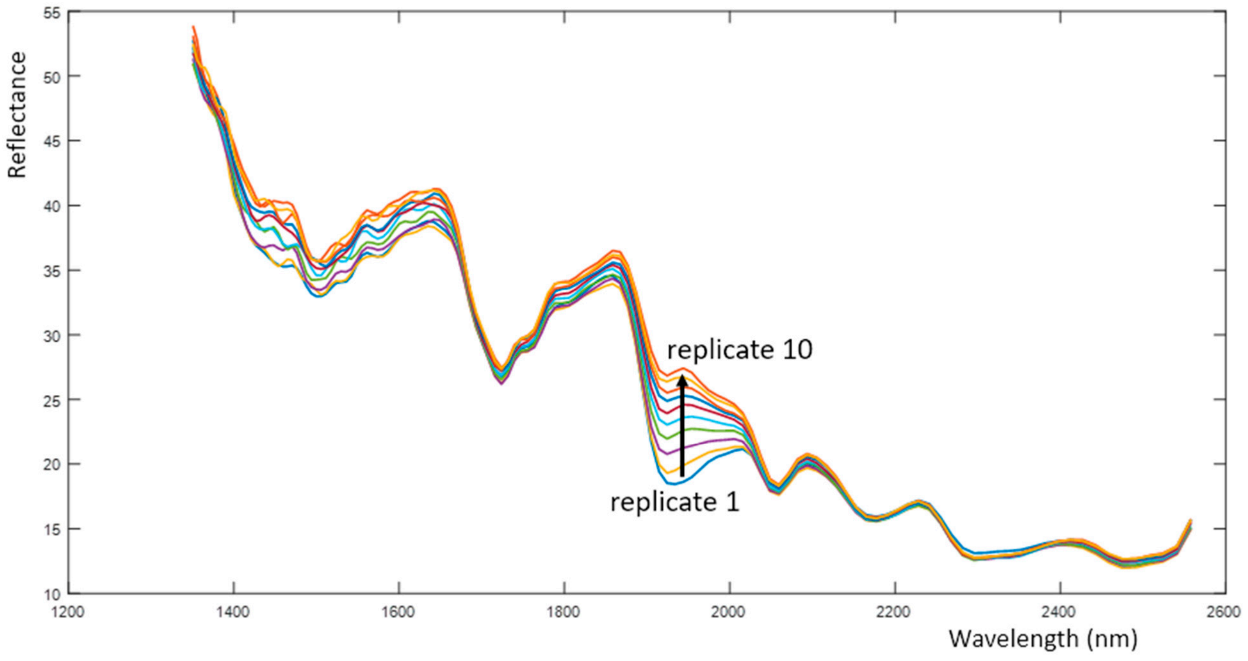

Figure S1. Ten instrumental replicates of a random A1 sample without sample repositioning. The band around 1950 nm shows a loss of humidity on consecutive replicates, probably due to the high temperature of the measurement window. The sample had been opened 10 days before and stored in room conditions.

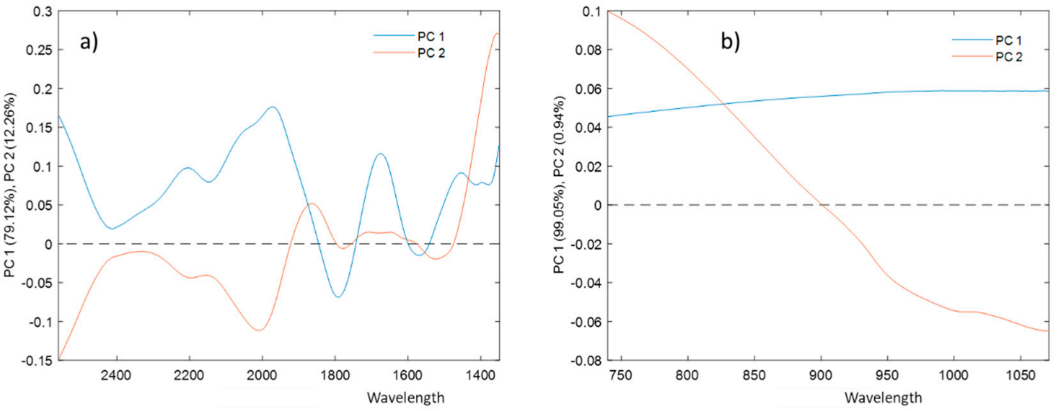

Figure S2. Loading plots for the two first PCs obtained with the PCA models using the a) NeoSpectra MDK and b) SCiO data.

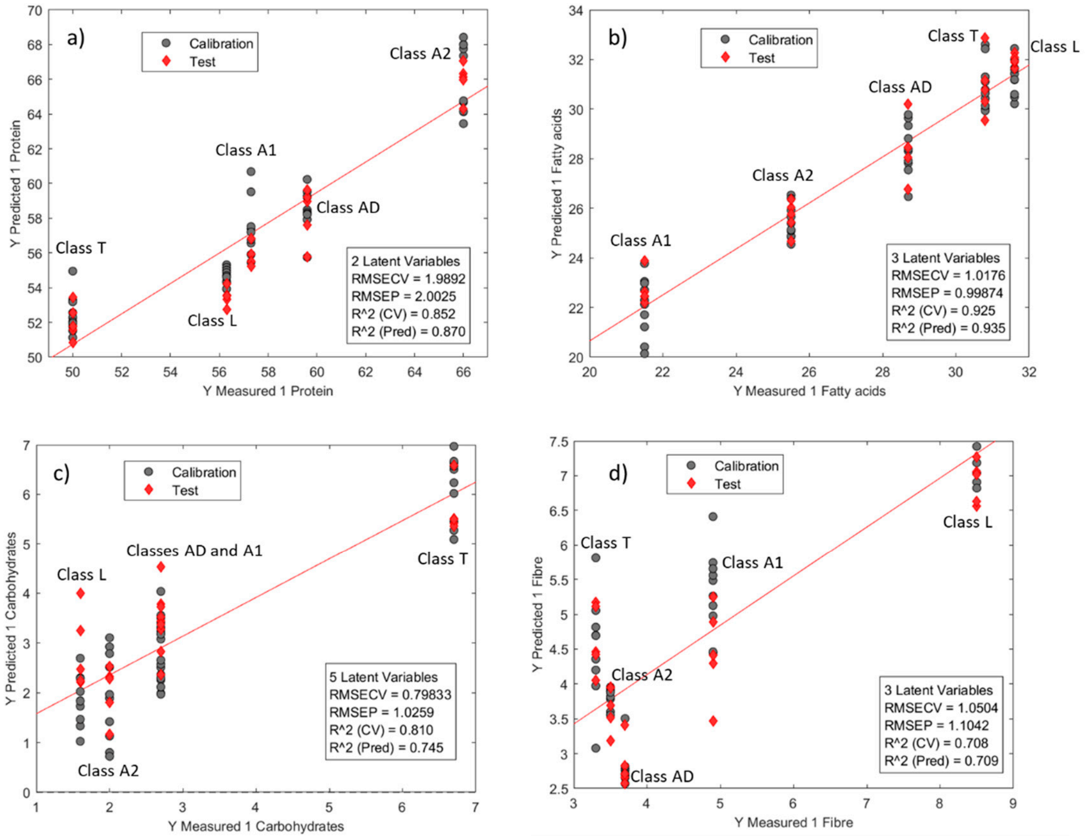

Figure S3. Regression lines between the predicted and the measured values for the different PLS models made with the NeoSpectra data. a) Protein, b) fatty acids, c) carbohydrates, d) fibre

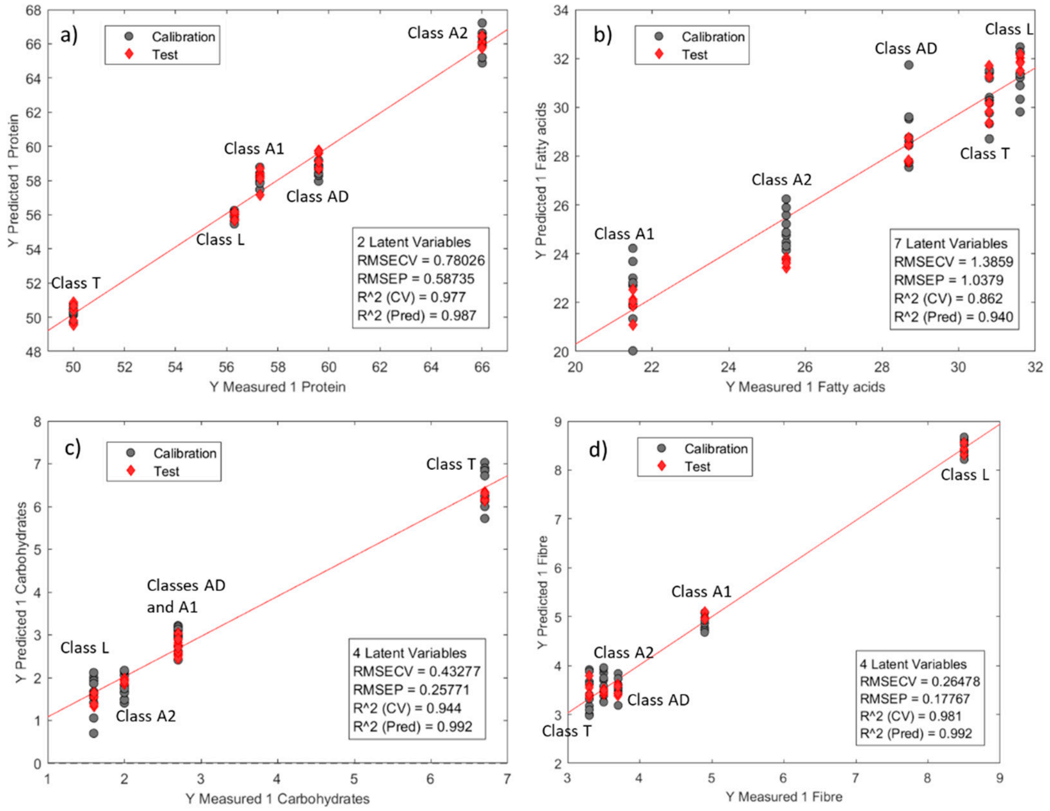

Figure S4. Regression lines between the predicted and the measured values for the different PLS models made with the SCiO data. a) Protein, b) fatty acids, c) carbohydrates, d) fibre
